# Overall and Sex-Specific Associations of Serum Lipid-Soluble Micronutrients with Metabolic Dysfunction-Associated Steatotic Liver Disease among Adults in the United States

**DOI:** 10.3390/nu16081242

**Published:** 2024-04-22

**Authors:** Weiwen Chai, Meng-Hua Tao

**Affiliations:** 1 Department of Nutrition and Health Sciences, University of Nebraska-Lincoln, 1700 N 35th Street, Lincoln, NE 68583, USA; 2 Department of Public Health Science, Henry Ford Health System, One Ford Place 3E, Detroit, MI 48202, USA; mtao1@hfhs.org

**Keywords:** serum lipid-soluble micronutrients, metabolic dysfunction-associated steatotic liver disease (MASLD), α-tocopherol, γ-tocopherol, 25-hydroxy-vitamin D, carotenoids, men, women

## Abstract

This study examined overall and sex-specific associations of serum lipid-soluble micronutrients including α- and γ-tocopherols, 25-hydroxy-vitamin D (25(OH)D), retinol, and six major carotenoids with metabolic dysfunction-associated steatotic lever disease (MASLD) using the 2017–2018 National Health and Nutrition Examination Survey. This analysis included 3956 adults (1991 men, 1965 women) aged ≥ 20 years. Steatotic liver disease was determined through transient elastography examination. Odds ratios (ORs) and 95% confidence intervals (95% CIs) for MASLD associated with micronutrients were estimated using logistic regressions. Higher serum α-tocopherol (highest vs. lowest quartile: OR = 1.53, 95% CI = 1.05–2.22, *p* = 0.03) and γ-tocopherol (highest vs. lowest quartile: OR = 4.15, 95% CI = 3.00–5.74, *p* < 0.0001) levels were associated with increased odds of MASLD. Higher serum 25(OH)D levels were associated with reduced odds of MASLD (highest vs. lowest quartile: OR = 0.41, 95% CI = 0.27–0.61, *p* = 0.0001). Inverse associations with the condition were also observed for carotenoids (α-carotene, β-carotene, α-cryptoxanthin, β-cryptoxanthin, combined lutein and zeaxanthin, and lycopene) in the serum (*Ps* < 0.05). The results were comparable between men and women, except for those on α-tocopherol, for which a positive association was only observed for men (*p* = 0.01). Our results suggest potential protective associations of serum 25(OH)D and carotenoids with MASLD. The positive associations between tocopherols and MASLD may reflect pathophysiological conditions associated with the condition.

## 1. Introduction

Nonalcoholic fatty liver disease (NAFLD) includes a broad range of liver conditions that are not alcohol-related [1]. While the nomenclature is widely used, there has been concerns that the term “nonalcoholic” does not accurately reflect the etiology of the disease [2]. In fact, it has been suggested that the underlying mechanisms for both NAFLD and alcohol-related liver disease could be overlapping [2]. Thus, a multi-stakeholder effort (Delphi process) led by three large pan-national liver associations developed a consensus for replacing NAFLD with the new nomenclature of metabolic dysfunction-associated steatotic liver disease (MASLD) with the presence of at least one of five cardiometabolic risk factors [2].

It has been suggested that insulin resistance and oxidative stress could be the key contributing factors to the pathogenesis of steatotic liver diseases (SLDs) [3,4,5]. Lipid-soluble micronutrients, such as tocopherols (forms of vitamin E), vitamin A, vitamin D, and carotenoids, play important roles in metabolism and maintaining tissue functions [6,7,8,9]. The majority of these micronutrients also function as antioxidants neutralizing free radicals and lowering oxidative stress [6,7,8,9]. In our recently published research, we assessed the dietary intake of lipid-soluble micronutrients with hepatic steatosis and observed that dietary α-tocopherol (the predominant form of vitamin E) and β-carotene intakes were inversely associated with the odds of having the disease condition [10]. However, micronutrients in the diet may not be directly reflected in blood and tissue content. Furthermore, research suggests that sex is an important factor associated with hepatic physiology and pathology [11]. Our previous study also found significantly higher rates of hepatic steatosis in men than in women [10]. Therefore, based on the criteria for the new nomenclature and definition of MASLD, the current study examined the overall and sex-specific associations of serum levels of lipid-soluble micronutrients including α-tocopherol, γ-tocopherol, retinol (vitamin A), 25-hydroxy-vitamin D (25(OH)D), and six major carotenoids (α-carotene β-carotene, α-cryptoxanthin, β-cryptoxanthin, combined lutein and zeaxanthin, and lycopene) with MASLD among adults in the United States, utilizing 2017–2018 Health and Nutrition Examination Survey (NHANES) data. We also sought to determine whether alcohol consumption would influence the relations between serum lipid-soluble micronutrients and MASLD.

## 2. Materials and Methods

### 2.1. Study Population

NHANES is an ongoing program of studies to assess the health and nutritional status in a nationally representative sample of the civilian, non-institutionalized U.S. population with its complex, multistage probability sampling design [12]. The NHANES 2017–2018 data cycle was used in the study as it includes the liver ultrasound transient elastography examinations that provide objective measures for SLD [12]. The analysis included participants aged 20 years or above. We further excluded participants who were pregnant or breastfeeding and participants with missing data on liver ultrasound transient elastography examination, education, and values of key criteria of metabolic dysfunction such as body mass index (BMI), waist circumference (WC), HDL-cholesterol (HDL), hemoglobin A1C (HbA1C), blood pressure measures (both systolic and diastolic blood pressure measures), history of diabetes, and history of hypertension. NHANES is conducted and maintained by the National Center for Health Statistics (NCHS). Institutional Review Board/Ethic Review Board of NCHS approved NHANES protocol (Continuation of Protocol #2011-17, effective through 26 October 2017; Protocol #2018-01, effective beginning from 26 October 2017). Written informed consent was obtained from all participants.

### 2.2. Serum Levels of Lipid-Soluble Micronutrients

Blood was drawn from participants in the NHANES Mobile Examination Center (MEC). Serum levels of α-tocopherol, γ-tocopherol, retinol, and carotenoids were measured using a modification of high-performance liquid chromatography with photodiode array detection method and spectrophotometric methods were used for quantitative analysis. The concentration of an unknown analyte was determined by comparing the peak height/peak area of the analyte in the unknown with the peak height/peak area of a known amount of the same analyte in a calibrator solution [13]. Total β-carotene levels were determined through the sum of cis and trans β-carotene. Serum 25(OH)D_2_ and 25(OH)D_3_ were measured using high-performance liquid chromatography-tandem mass spectrometry (HPLC-MS/MS). Quantitation was estimated via comparing the response ratio (peak area of the analyte/peak area of the internal standard) of the unknown with the response ratio of a known amount of analyte in a calibrator solution [14]. Total serum vitamin D (25(OH)D) concentration was the sum of concentrations of 25(OH)D_2_ and 25(OH)D_3_ in the serum.

### 2.3. Defining Steatotic Liver Disease (SLD) and Its Subtypes

To define SLD, we used the measures from the liver ultrasound transient elastography performed in the NHANES MEC using the FibroScan^®^ device (Echosens, Cambridge, MA, USA). The device includes a novel physical parameter, controlled attenuation parameter (CAP^TM^) (Echosens, Cambridge, MA, USA), which measures the ultrasound attenuation related to the presence of SLD. A minimum of a three-hour fast was required of all participants before the examination. The above procedure is considered reliable and non-invasive and has been detailed in the NHANES Liver Ultrasound Transient Elastography Procedures manual [15]. Several studies that assessed the accuracy of CAP^TM^ measurement against biopsy reported 76–79%, 71–79%, and ≥80% for sensitivity, specificity, and area under ROC curve, respectively [16,17,18]. We used 302 dB/m (>5% steatosis) as the threshold value to define participants with SLD based on a prospective study [19].

In the presence of SLD, the finding of any level of a cardiometabolic risk factor would be considered as indicating MASLD if the participant met at least one of the following cardiometabolic risk criteria: (1) BMI > 25 kg/m^2^ or WC > 94 cm (male [M]) or >80 cm (Female [F]); (2) HbA1c > 5.7% or having history of diabetes; (3) blood pressure ≥ 130/85 mmHg or having history of high blood pressure; (4) plasma HDL levels ≤ 40 mg/dL (M) or ≤50 mg/dL (F); and (5) triglyceride (TG) level ≥ 150 mg/dL [2,20].

Delphi process has also created an additional category (separated from pure MASLD), namely MetALD, for those having MASLD with moderate alcohol consumption (F: 140 to 350 g/week or 20–50 g/day; M: 210 to 420 g/week or 30–60 g/day) [2,20]. Daily alcohol intake was obtained from the 24 h dietary recall conducted in NHANES 2017–2018 using the USDA’s Automated Multiple-Pass Method [21,22]. Two 24 h recalls were conducted; the first was collected in person by trained interviewers and the second was completed by trained interviewers via telephone 3–10 days after the first interview [21]. We used the first dietary recall since it was performed in person by trained interviewers. There were significant correlations of alcohol intake between the first and second dietary recalls (*p* < 0.0001)

### 2.4. Statistical Analyses

Odds ratios (ORs) and 95% confidence intervals (95% CIs) for associations for micronutrients with MASLD were estimated using logistic regressions (proc survey logistic). Serum levels of micronutrients (α-tocopherol, γ-tocopherol, retinol, 25(OH)D, and six major carotenoids) were categorized into quartiles. The lowest category was used as the reference. P for trend (test for dose-response effects) was estimated to determine whether ORs of MASLD increased or decreased in magnitude with higher levels/quartiles of micronutrients. For overall associations, the model was adjusted for age, sex, ethnicity (black, Hispanic, white, or Asian/other), education (less than high school, high school/some college, or college graduate), smoking status (never, former, or current smoker), daily alcohol consumption (from 24 h dietary recall), and alcohol drinking habits defined based on the question “Past 12 months how often have alcohol drink”. For γ-tocopherol, the model was further adjusted for serum high-sensitive C-reactive protein (HS-CRP) levels. For sex-specific associations, we repeated the above analyses among men and women. Based on Delphi definition, pure MASLD was defined as MASLD with alcohol consumption < 20 g/day for F and <30 g/day for M and MetALD was defined as MASLD with concomitant moderate alcohol consumption (F: 20–50 g/day; M: 30–60 g/day) [2,19]. Thus, we also repeated the above analyses, stratified by participants’ alcohol consumption: (1) low alcohol consumption (F: <20 g/day; M: <30 g/day) and (2) moderate alcohol consumption (F: 20–50 g/day; M: 30–60 g/day).

Furthermore, we assessed correlations between serum and dietary levels of individual micronutrients (α-tocopherol, vitamin D, retinol, α-carotene, β-carotene, β-cryptoxanthin, combined lutein and zeaxanthin, and lycopene) using Spearman correlation coefficients. The daily intakes of dietary micronutrients were estimated from 24 h recall. The method for determining dietary micronutrients was described in our previously published research [10]. Correlations were not assessed for γ-tocopherol and α-cryptoxanthin because no direct data were available for dietary intake of these two micronutrients. The ‘Survey’ procedure in SAS 9.4 software (SAS Institute, Cary, NC, USA) was used, accounting for the complex, multistage, clustered probability sampling design of the NHANES. All tests were two-sided, and *p* < 0.05 was used as the critical value for statistical significance.

## 3. Results

A total of 3956 participants (1991 men, 1965 women) were involved in the final analytic sample. The prevalence of NASLD was 27.7% (34.1% among men and 21.2% among women). Overall, participants with NASLD were older and less likely to have college degrees compared to those without the condition (*Ps* < 0.05). Higher proportions of participants with NASLD were also found in Hispanics and former smokers (*Ps* < 0.05). Furthermore, participants with MASLD had higher BMI, WC, HbA1C, and TG values and lower HDL levels, as well as higher incidences of history of diabetes and higher blood pressure, compared to those without MASLD (*p* < 0.0001). Similar data were observed separately among men and women (Table 1).

The overall and sex-specific associations of serum micronutrients with MASLD are shown in Table 2. Higher serum α-tocopherol (highest vs. lowest quartile: OR = 1.53, 95% CI = 1.05–2.22, *P*_trend_ = 0.03 and γ-tocopherol (highest vs. lowest quartile: OR = 4.15, 95% CI = 3.00–5.74, *P*_trend_ < 0.0001) levels were associated with increased odds of MASLD. Higher serum 25(OH)D levels were associated with reduced odds of MASLD (highest vs. lowest quartile: OR = 0.41, 95% CI = 0.27–0.61, *P*_trend_ = 0.0001). Inverse associations with MASLD were also observed for carotenoids such as α-carotene (*P*_trend_ < 0.0001), β-carotene (*P*_trend_ < 0.0001), α-cryptoxanthin (*P*_trend_ < 0.0001), β-crypotoxanthin (*P*_trend_ = 0.008), combined lutein and zeaxanthin (*P*_trend_ < 0.0001), and lycopene (*P*_trend_ = 0.02) in the serum. For γ-tocopherol, results remained significant after the further adjustment of serum HS-CRP concentrations (highest vs. lowest quartile: OR = 3.19, 95% CI = 2.30–4.42, *P*_trend_ < 0.0001).

The results observed in men and women were comparable to the overall results (for all participants). Positive associations of γ-tocopherol and inverse associations of 25(OH)D and carotenoids (α-carotene, β-carotene, α-cryptoxanthin, β-cryptoxanthin, and combined lutein and zeaxanthin) with odds of MASLD were found in both men and women. However, serum α-tocopherol levels were positively associated with odds of MASLD in men (*P*_trend_ = 0.01) and no association was observed in women (*P*_trend_ = 0.67).

After stratification by alcohol consumption, similar trends of associations of serum micronutrients with MASLD were observed, both for participants with low alcohol consumption (F: <20 g/d; M: <30 g/d) as well as for participants with moderate alcohol consumption (F: 20–50 g/d; M: 30–60 g/d). A positive association was found for serum γ-tocopherol levels for both low (*P*_trend_ < 0.0001) and moderate alcohol consumers (*P*_trend_ < 0.0001). Inverse associations were also observed for α-carotene and β-carotene for both subgroups (*Ps* < 0.01). Inverse associations of serum 25(OH)D, α-cryptoxanthin, β-cryptoxanthin, combined lutein and zeaxanthin, and lycopene were found among low alcohol consumers (*Ps* < 0.005) but not among moderate alcohol consumers (*Ps* > 0.05). For retinol, an inverse association with MASLD was observed among individuals with moderate alcohol intake (*P*_trend_ = 0.004) but not among individuals with low alcohol consumption (*P*_trend_ = 0.20) (Table 3).

Table 4 shows both correlations between dietary intake and serum levels of individual micronutrients as well as mean values of micronutrients from the diet and in the serum. Overall, the correlations between dietary and serum levels of micronutrients appeared to be higher for carotenoids (α-carotene, r = 0.34; β-carotene, r = 0.31; β-cryptoxanthin, r = 0.29; combined lutein and zeaxanthin, r = 0.30; lycopene; r = 0.25) than for other lipid-soluble micronutrients (α-tocopherol, r = 0.10; retinol, r = 0.05; vitamin D, r = 0.16). Similar patterns were observed in participants with and without MASLD. The mean levels of these lipid-soluble micronutrients in the serum did not reach the thresholds for deficiency or toxicity [23].

## 4. Discussion

Utilizing the newly developed criteria that define MASLD and the more recent NHANES cycle that had transient elastography measures available for objectively detecting both SLD as well as values of the serum concentrations of major lipid-soluble micronutrients, the current results showed that higher serum α-tocopherol and γ-tocopherol levels (forms of vitamin E) were associated with increased odds of MASLD. On the other hand, higher serum 25(OH)D (indicator of vitamin D status) and carotenoid levels were associated with reduced odds of MASLD. We previously assessed associations between the dietary intakes of major lipid-soluble micronutrients and hepatic steatosis with the adjustment of most of the cardiometabolic risk factors (history of diabetes, history of hypertension, BMI, etc.) [10]. Thus, in this study, we further examined the associations of serum levels of lipid-soluble micronutrients with newly defined MASLD, which would provide a more complete picture for understanding the relationships between micronutrients and the disease condition.

Vitamin D has been suggested as an important physiological regulator beyond its classical role in bone and calcium homeostasis [24]. Our results were in agreement with previous observational studies that reported that individuals with NAFLD had lower levels of serum 25(OH)D compared to those without NAFLD [25,26,27]. One study that utilized NHANES III (1988–1994) data reported that serum 25(OH)D levels were independently and inversely associated with the severity of NAFLD [28]. Our results on carotenoids were also consistent with those from a previous study by Christensen et al. that reported that serum carotenoids such as α-carotene, β-carotene, β-cryptoxanthin, and combined lutein and zeaxanthin were inversely associated with the odds of NAFLD using NHANES 2003–2014 data although the liver steatosis status was not determined using the objective transient elastography measures in that study [29]. Our previous research found no associations of dietary vitamin D intake with steatosis and an inverse association between dietary β-carotene and steatosis [10]. With the exception that a person takes high dosages of vitamin D supplements, dietary vitamin D is not the main source of vitamin D in the body [30]. In our study, there was only a 16% correlation between dietary vitamin D intake and serum vitamin D (25(OH)D) levels while a higher correlation (31%) was found for dietary and serum β-carotene levels.

α-tocopherol (the main form of vitamin E) has been suggested as a potential treatment for liver diseases because of its anti-oxidative functions [1]. Several randomized clinical trials demonstrated significant improvements in liver histology with vitamin E treatment compared to placebos [31,32]. In our previous analysis using the same NAHNES cycle (2017–2018), we found that a higher dietary vitamin E intake was associated with reduced odds of hepatic steatosis [10]. However, in this study, we observed that serum α-tocopherol levels were positively associated with the odds of the newly defined MASLD, particularly in men, which was contradictory to our previous findings on dietary vitamin E and the disease condition. This may suggest that circulating vitamin E (α-tocopherol) may not directly reflect an individual’s dietary vitamin E (as α-tocopherol). In fact, we found a low correlation (10%) between dietary and serum α-tocopherol levels. Thus, higher circulating α-tocopherol levels observed in individuals with MASLD relative to those without the condition may suggest a pathophysiological condition associated with the disease and a poor health status in general. Our previous study also found that serum α-tocopherol levels were positively associated with all-cause mortality using NHANES 1999–2002 data [33].

In our study, we observed that higher serum γ-tocopherol levels were associated with increased odds of MASLD. Although no previous studies have examined associations of circulating γ-tocopherol with SLD, our results were consistent with studies that assessed relations between serum γ-tocopherol and other disease conditions. For example, Chai et al. reported that serum γ-tocopherol was positively associated with all-cause, cancer, and cardiovascular disease mortality in a multiethnic population [34]. In animals and cultured fibroblasts, γ-tocopherol levels increase to respond to inflammatory signals [35,36]. The association between γ-tocopherol in the serum and MASLD remained significant after further adjusting for HS-CRP (indicator for chronic inflammation) in the model, suggesting a potential role for γ-tocopherol as a biomarker (independent of other inflammatory biomarkers such as CRP) in response to pathological conditions.

Sex is thought to play a key role in liver [11] and other metabolic diseases [37,38]. In this study, the prevalence of MASLD was significantly higher in men (34.1%) than in women (21.2%). Thus, sex-specific prevention and treatment strategies appear to be necessary to reduce the incidence of MASLD. In our analyses stratified by sex, we found that men and women had similar results in terms of associations of serum micronutrients with MASLD except for α-tocopherol, for which a significant association was observed only in men. Our study also found that age was another important contributor to MASLD as participants with MSALD were older than those without the disease in both men and women. Therefore, we adjusted for participant’s age (as a continuous variable/covariate) in our analysis models to remove the potential influence of age on the relationships between micronutrients and MASLD.

The newly defined MASLD reflects the strong epidemiological and pathogenic link between NAFLD, metabolic dysfunction, and insulin resistance. In terms of the role of alcohol drinking in SLD, it is now acknowledged that biological mechanisms contributing to both NAFLD and alcohol-related liver disease (ALD) could be overlapping. We adjusted for participants’ daily alcohol intake and drink habits in our analyses and also performed stratified analysis based on the Delphi definitions [2] of low (F: <20 g/day; M: <30 g/day) and moderate alcohol consumption (F: 20–50 g/day; M: 30–60 g/day). The trends of the associations of serum micronutrients with MASLD were similar for the majority of the micronutrients (e.g., γ-tocopherol, 25(OH)D, α-carotene, β-carotene, α-cryptoxanthin, β-cryptoxanthin, combined lutein and zeaxanthin) for both subgroups (low alcohol vs. moderate alcohol consumers). Some of the associations among moderate alcohol consumers were not statistically significant, possibly due to the small sample size of this subgroup. Thus, our results suggest that alcohol consumption may not significantly influence the relationships between serum lipid-soluble micronutrients and MASLD.

The correlations between the dietary intake of lipid-soluble micronutrients and their concentrations in the circulation have been studied previously. For example, serum/plasma levels of carotenoids are determined not only by an individual’s dietary intake of carotenoids but also by other physiologic factors. Research has shown that carotenoids levels are lower among obese individuals compared to their normal-weight counterparts [39], possibly due to the higher amount of subcutaneous fat and increased oxidative stress associated with obesity [40]. In our study, we found that the correlations between dietary and serum levels of micronutrients appeared to be higher for carotenoids (α-carotene, r = 0.34; β-carotene, r = 0.31; β-cryptoxanthin, r = 0.29; combined lutein and zeaxanthin, r = 0.30; lycopene; r = 0.25) than for other micronutrients such as α-tocopherol (r = 0.10), retinol (r = 0.05), and 25(OH)D (r = 0.16), suggesting that serum levels of carotenoids partially reflect their dietary intake. The correlations between dietary intake and serum levels of micronutrients were consistent for participants with and without MASLD. This may partly explain the fact that we observed protective associations with SLD for both dietary [10] and serum β-carotene while a protective association was observed for dietary vitamin E (α-tocopherol) [10] but not for serum α-tocopherol, for which a positive association was found in men.

To our knowledge, the current study was the first to investigate associations between major lipid-soluble micronutrients in the serum and newly defined MASLD using a representative sample of the U.S. population. One main strength of the study was the utilization of liver ultrasound transient elastography, an objective measure for SLD. Our study had limitations. The cross-sectional study design may not have determined the temporal sequences. The daily alcohol consumption (g/d) was obtained from the one-time 24 h dietary recall, which may not have completely captured the alcohol exposure of the participants. However, the data on alcohol intake from 24 h dietary recalls corresponded to the alcohol drinking habits of the participants. The mean alcohol consumptions from the 24 h dietary recalls were 0.3 g/d, 4.1 g/d, 13.6 g/d, and 40.0 g/d for never/rare, occasional, sometimes, and frequent alcohol drinkers, respectively.

## 5. Conclusions

In conclusion, our study found that higher serum α-tocopherol and γ-tocopherol levels were associated with increased odds of MASLD. Higher serum 25(OH)D and carotenoid levels were associated with reduced odds of having the condition. In general, the associations of lipid-soluble micronutrients with MASLD were comparable both between men and women as well as between those with low and moderate alcohol consumption. Our results suggest potential protective associations of serum lipid-soluble micronutrients such as 25(OH)D and carotenoids with MASLD whereas the positive associations between tocopherols and MASLD may indicate potential pathophysiological conditions associated with the condition. Due to the cross-sectional nature, our results should be confirmed by future longitudinal studies.

## Figures and Tables

**Table 1:** Characteristics of study participants, divided by MASLD status and sex.

	All Participants			Men			Women		
	Non-MASLD	MASLD	*p* ^a^	Non-MASLD	MASLD	*p* ^a^	Non-MASLD	MASLD	*p* ^a^
N	2808	1148		1304	687		1504	461	
MASLD prevalence (%)		27.7			34.1			21.2	
Age (y)	46.7 ± 0.7	51.4 ± 0.7	<0.0001	45.2 ± 0.8	51.0 ± 0.8	<0.0001	48.0 ± 0.8	52.1 ± 0.9	<0.0001
Sex, (%)			<0.0001						
Men	45.7	61.8							
Women	54.3	38.2							
Ethnicity (%)			0.002			<0.0001			0.29
Black	11.4	7.6		11.6	6.2		11.1	9.7	
White	63.5	64.3		62.2	65.5		64.5	62.5	
Hispanic	14.3	18.7		14.7	19.0		14.1	18.3	
Asian and others	10.8	9.4		11.5	9.3		10.3	9.5	
Education (%)			0.006			0.03			0.13
Below high school	10.3	10.5		10.9	11.4		9.7	9.0	
High school/some college	56.6	63.9		56.0	63.4		57.1	64.9	
College graduate	33.2	25.6		33.1	25.2		33.2	26.1	
Body mass index (kg/m^2^)	27.6 ± 0.3	34.9 ± 0.5	<0.0001	27.3 ± 0.3	33.9 ± 0.5	<0.0001	27.9 ± 0.4	36.3 ± 0.7	<0.0001
Waist circumference (cm)	95.2 ± 0.6	114.8 ± 1.1	<0.0001	96.9 ± 0.6	115.4 ± 1.2	<0.0001	93.9 ± 0.9	113.9 ± 1.5	<0.0001
HDL (mg/dL)	56.2 ± 0.6	46.3 ± 0.7	<0.0001	50.5 ± 0.4	43.1 ± 0.6	<0.0001	60.9 ± 0.9	51.4 ± 1.0	<0.0001
LDL (mg/dL)	110.5 ± 1.7	113.3 ± 2.8	0.15	109.8 ± 2.1	112.3 ± 3.1	0.08	111.1 ± 2.4	114.6 ± 4.0	0.09
Triglycerides (mg/dL)	99.4 ± 2.2	150.0 ± 9.3	<0.0001	108.2 ± 4.6	164.7 ± 13.7	0.0001	90.9 ± 3.4	129.0 ± 6.4	0.007
Hemoglobin A1C (%)	5.50 ± 0.02	6.08 ± 0.04	<0.0001	5.50 ± 0.03	6.04 ± 0.05	<0.0001	5.50 ± 0.02	6.15 ± 0.08	<0.0001
Smoke status (%)			<0.0001			0.006			0.01
Never	58.8	51.1		49.2	46.1		66.9	59.3	
Former	22.6	32.8		29.3	38.6		17.0	23.4	
Current	18.5	16.1		21.5	15.3		16.1	17.3	
Alcohol drinking habits in past 12 months (%)			0.24			0.24			0.05
Never/rarely	30.9	35.6		27.4	34.0		33.9	38.3	
Occasionally	21.3	23.2		18.2	18.9		23.9	30.2	
Sometimes	32.9	29.3		34.2	30.8		31.8	26.8	
Frequently	14.9	11.9		20.2	16.3		10.3	4.7	
Alcohol intake (g/d) ^b^	12.1 ± 0.7	10.6 ± 1.4	0.79	16.4 ± 1.3	13.9 ± 1.8	0.65	8.4 ± 0.6	5.3 ± 1.2	0.35
Diabetes (%)	8.2	26.9	<0.0001	8.3	29.1	<0.0001	8.2	23.4	<0.0001
High blood pressure (%)	24.6	48.8	<0.0001	29.4	52.1	<0.0001	26.3	61.2	<0.0001

Note: Values are presented as weighted means ± SEs and weighted percentages (%). MASLD = Metabolic dysfunction-associated steatotic liver disease. ^a^ *p* values for differences between participants with NASLD and participants without the condition, using *t*-tests for continuous variables and chi-squared tests for categorical variables. ^b^ From 24 h dietary recall.

**Table 2:** Overall and sex-specific associations of serum levels of micronutrients with MASLD.

	All Participants		Men		Women	
Quartile (Q1–Q4)	OR (95% CI) ^a^	*P*_trend_ ^a^	OR (95% CI) ^b^	*P*_trend_ ^b^	OR (95% CI) ^b^	*P*_trend_ ^b^
α-tocopherol (µg/dL)						
Q1 (<858)	1.00		1.00		1.00	
Q2 (858–1050)	1.08 (0.81–1.46)		1.05 (0.70–1.57)		1.11 (0.68–1.81)	
Q3 (1050–1310)	1.55 (1.13–2.11)		1.54 (1.09–2.16)		1.43 (0.93–2.21)	
Q4 (≥1310)	1.53 (1.05–2.22)	0.03	1.79 (1.21–2.65)	0.01	1.21 (0.72–2.05)	0.67
γ-tocopherol (µg/dL)						
Q1 (<112)	1.00		1.00		1.00	
Q2 (112–157)	1.23 (0.86–1.77)		0.92 (0.56–1.51)		2.14 (1.14–3.90)	
Q3 (157–214)	1.93 (1.29–2.89)		1.26 (0.82–1.93)		3.94 (1.86–8.35)	
Q4 (≥214)	4.15 (3.00–5.74)	<0.0001	2.95 (1.93–4.51)	<0.0001	7.36 (4.45–12.18)	<0.0001
25(OH)D (nmol/L)						
Q1 (<47.4)	1.00		1.00		1.00	
Q2 (47.4–63.7)	0.65 (0.46–0.93)		0.58 (0.39–0.85)		0.77 (0.41–1.45)	
Q3 (63.7–81.6)	0.51 (0.36–0.74)		0.51 (0.34–0.75)		0.49 (0.28–0.86)	
Q4 (≥81.6)	0.41 (0.27–0.61)	0.0001	0.43 (0.30–0.60)	<0.0001	0.40 (0.20–0.79)	0.003
Retinol (µg/dL)						
Q1 (<38.1)	1.00		1.00		1.00	
Q2 (38.1–46.9)	0.96 (0.64–1.43)		1.05 (0.65–1.71)		0.96 (0.56–1.65)	
Q3 (46.9–57.8)	1.08 (0.79–1.47)		1.23 (0.82–1.85)		1.09 (0.64–1.87)	
Q4 (≥57.8)	1.05 (0.69–1.60)	0.62	1.28 (0.77–2.14)	0.26	0.97 (0.52–1.83)	0.99
α-carotene (µg/dL)						
Q1 (<1.4)	1.00		1.00		1.00	
Q2 (1.4–2.7)	0.74 (0.51–1.08)		0.94 (0.57–1.54)		0.55 (0.32–0.93)	
Q3 (2.7–5.5)	0.36 (0.26–0.50)		0.44 (0.27–0.71)		0.28 (0.20–0.39)	
Q4 (≥5.5)	0.24 (0.18–0.33)	<0.0001	0.28 (0.18–0.43)	<0.0001	0.20 (0.15–0.28)	<0.0001
β-carotene (µg/dL)						
Q1 (<7.7)	1.00		1.00		1.00	
Q2 (7.7–13.2)	0.46 (0.35–0.62)		0.45 (0.29–0.70)		0.48 (0.32–0.72)	
Q3 (13.2–23.8)	0.30 (0.21–0.43)		0.31 (0.20–0.48)		0.29 (0.20–0.42)	
Q4 (≥23.8)	0.16 (0.11–0.24)	<0.0001	0.17 (0.09–0.34)	<0.0001	0.16 (0.10–0.25)	<0.0001
α-cryptoxanthin (µg/dL)						
Q1 (<1.8)	1.00		1.00		1.00	
Q2 (1.8–2.6)	0.72 (0.55–0.96)		0.79 (0.55–1.13)		0.67 (0.46–0.97)	
Q3 (2.6–3.8)	0.47 (0.34–0.66)		0.51 (0.34–0.76)		0.43 (0.26–0.73)	
Q4 (≥3.8)	0.28 (0.20–0.38)	<0.0001	0.46 (0.28–0.76)	0.0005	0.12 (0.07–0.20)	<0.0001
β-cryptoxanthin (µg/dL)						
Q1 (<4.6)	1.00		1.00		1.00	
Q2 (4.6–7.5)	0.86 (0.68–1.08)		0.86 (0.60–1.23)		0.87 (0.66–1.16)	
Q3 (7.5–13.0)	0.46 (0.33–0.65)		0.58 (0.41–0.83)		0.34 (0.22–0.51)	
Q4 (≥13.0)	0.51 (0.29–0.89)	0.008	0.75 (0.36–1.56)	0.36	0.30 (0.17–0.54)	<0.0001
Lutein and zeaxanthin (µg/dL)						
Q1 (<11.6)	1.00		1.00		1.00	
Q2 (11.6–16.3)	0.72 (0.52–0.99)		0.69 (0.40–1.20)		0.76 (0.40–1.46)	
Q3 (16.3–23.5)	0.63 (0.39–1.01)		0.67 (0.38–1.19)		0.57 (0.31–1.05)	
Q4 (≥23.5)	0.40 (0.29–0.56)	<0.0001	0.47 (0.25–0.90)	0.039	0.32 (0.21–0.50)	<0.0001
Lycopene (µg/dL)						
Q1 (<25.0)	1.00		1.00		1.00	
Q2 (25.0–35.8)	0.92 (0.63–1.33)		0.81 (0.48–1.37)		1.10 (0.72–1.66)	
Q3 (35.8–48.6)	0.94 (0.71–1.25)		0.77 (0.48–1.21)		1.22 (0.79–1.90)	
Q4 (≥48.6)	0.71 (0.50–0.99)	0.02	0.66 (0.41–1.06)	0.05	0.76 (0.45–1.27)	0.28

Note: MASLD = Metabolic dysfunction-associated steatotic liver disease. ^a^ Odds ratio (OR), 95% confidence interval (95% CI), and *P*_trend_ values were estimated via logistic regression (Proc Survey Logistic). Analyses were adjusted for age, sex, ethnicity, education, smoking status, alcohol drinking habits, and daily alcohol consumption. ^b^ Odds ratio (OR), 95% confidence interval (95% CI), and *P*_trend_ values were estimated via logistic regression (Proc Survey Logistic). Analyses were adjusted for age, ethnicity, education, smoking status, alcohol drinking habits, and daily alcohol consumption.

**Table 3:** Associations of serum levels of lipid-soluble micronutrients with MASLD, divided by alcohol consumption.

	Low Alcohol Consumption (M: <30 g/d; F: <20 g/d) ^a^ Cases/Participants = 932/3186		Moderate Alcohol Consumption (M: 30–60 g/d; F 20–50 g/d) ^a^ Cases/Participants = 74/296	
Quartile (Q1–Q4)	OR (95% CI) ^b^	*P*_trend_ ^b^	OR (95% CI) ^b^	*P*_trend_ ^b^
α-tocopherol (µg/dL)				
Q1 (<858)	1.00		1.00	
Q2 (858–1050)	1.06 (0.81–1.39)		1.28 (0.33–5.05)	
Q3 (1050–1310)	1.52 (1.07–2.15)		2.28 (0.58–8.99)	
Q4 (≥1310)	1.48 (1.00–2.20)	0.056	1.46 (0.46–4.65)	0.96
γ-tocopherol (µg/dL)				
Q1 (<112)	1.00		1.00	
Q2 (112–157)	1.25 (0.89–1.78)		2.62 (0.52–13.26)	
Q3 (157–214)	1.83 (1.21–2.78)		8.03 (1.59–40.49)	
Q4 (≥214)	4.13 (2.80–6.11)	<0.0001	8.84 (2.95–26.49)	<0.0001
25(OH)D (nmol/L)				
Q1 (<47.4)	1.00		1.00	
Q2 (47.4–63.7)	0.63 (0.44–0.91)		1.11 (0.22–5.55)	
Q3 (63.7–81.6)	0.52 (0.37–0.74)		1.14 (0.33–3.95)	
Q4 (≥81.6)	0.42 (0.28–0.63)	0.0004	0.41 (0.10–1.68)	0.11
Retinol (µg/dL)				
Q1 (<38.1)	1.00		1.00	
Q2 (38.1–46.9)	0.94 (0.68–1.31)		0.74 (0.18–3.08)	
Q3 (46.9–57.8)	1.13 (0.81–1.58)		0.34 (0.10–1.11)	
Q4 (≥57.8)	1.20 (0.78–1.84)	0.20	0.20 (0.05–0.70)	0.004
α-carotene (µg/dL)				
Q1 (<1.4)	1.00		1.00	
Q2 (1.4–2.7)	0.65 (0.44–0.96)		0.73 (0.25–2.16)	
Q3 (2.7–5.5)	0.33 (0.24–0.47)		0.19 (0.06–0.62)	
Q4 (≥5.5)	0.23 (0.16–0.33)	<0.0001	0.19 (0.06–0.61)	0.004
β-carotene (µg/dL)				
Q1 (<7.7)	1.00		1.00	
Q2 (7.7–13.2)	0.46 (0.35–0.59)		0.36 (0.11–1.18)	
Q3 (13.2–23.8)	0.31 (0.21–0.46)		0.13 (0.05–0.37)	
Q4 (≥23.8)	0.16 (0.11–0.24)	<0.0001	0.10 (0.03–0.41)	0.008
α-cryptoxanthin (µg/dL)				
Q1 (<1.8)	1.00		1.00	
Q2 (1.8–2.6)	0.77 (0.54–1.10)		0.51 (0.12–2.24)	
Q3 (2.6–3.8)	0.46 (0.31–0.67)		0.93 (0.17–5.09)	
Q4 (≥3.8)	0.28 (0.20–0.39)	<0.0001	0.17 (0.03–1.10)	0.16
β-cryptoxanthin (µg/dL)				
Q1 (<4.6)	1.00		1.00	
Q2 (4.6–7.5)	0.89 (0.68–1.16)		0.67 (0.22–2.04)	
Q3 (7.5–13.0)	0.41 (0.30–0.55)		0.58 (0.17–1.93)	
Q4 (≥13.0)	0.49 (0.29–0.85)	0.002	0.42 (0.11–1.70)	0.29
Lutein and zeaxanthin (µg/dL)				
Q1 (<11.6)	1.00		1.00	
Q2 (11.6–16.3)	0.70 (0.51–0.97)		0.85 (0.18–3.93)	
Q3 (16.3–23.5)	0.63 (0.37–1.07)		0.57 (0.25–1.28)	
Q4 (≥23.5)	0.38 (0.28–0.52)	<0.0001	0.39 (0.10–1.47)	0.12
Lycopene (µg/dL)				
Q1 (<25.0)	1.00		1.00	
Q2 (25.0–35.8)	1.04 (0.69–1.56)		0.62 (0.21–1.82)	
Q3 (35.8–48.6)	1.03 (0.75–1.40)		0.46 (0.17–1.27)	
Q4 (≥48.6)	0.68 (0.50–0.94)	0.002	0.92 (0.37–2.33)	0.90

Note: MASLD = Metabolic dysfunction-associated steatotic liver disease. ^a^ From 24 h dietary recall. ^b^ Odds ratio (OR), 95% confidence interval (95% CI), and *P*_trend_ values were estimated via logistic regression (Proc Survey Logistic). Analyses were adjusted for age, sex, ethnicity, education, and smoking status.

**Table 4:** Dietary intake and serum levels of lipid-soluble micronutrients and their correlations.

		α-Toc	Vit. D	Retinol	α-Carot	β-Carot	β-Cryp	Lut/zea	Lyco
All participants	Correlation Coefficient (r) ^a^	0.10	0.16	0.05	0.34	0.31	0.29	0.30	0.25
	Dietary Intake (µg/d)	9.6 ± 0.2 (mg/d)	4.2 ± 0.1	418 ± 9	396 ± 43	2535 ± 159	89.4 ± 7.0	1694 ± 114	5180 ± 253
	Serum (µg/dL)	1247 ± 11	73.3 ± 1.7 (nmol/L)	54.2 ± 0.3	5.3 ± 0.3	20.9 ± 0.9	9.1 ± 0.4	19.7 ± 0.6	40.4 ± 0.6
MASLD ^b^	Correlation Coefficient ^a^	0.09	0.17	0.11	0.30	0.31	0.29	0.25	0.24
	Dietary Intake (µg/d)	9.2 ± 0.3 (mg/d)	4.3 ± 0.1	415 ± 22	416 ± 70	2449 ± 214	90.3 ± 6.8	1328 ± 97	5006 ± 378
	Serum (µg/dL)	1314 ± 26	69.5 ± 2.3 (nmol/L)	55.5 ± 0.8	3.6 ± 0.3	13.6 ± 0.5	7.9 ± 0.5	17.5 ± 0.8	38.1 ± 1.1
Non-MASLD ^c^	Correlation Coefficient ^a^	0.07	0.15	0.11	0.36	0.34	0.29	0.32	0.26
	Dietary Intake (µg/d)	9.8 ± 0.3 (mg/d)	4.1 ± 0.2	419 ± 12	388 ± 42	2568 ± 181	89.0 ± 9.0	1835 ± 145	5247 ± 259
	Serum (µg/dL)	1222 ± 13	74.6 ± 1.7 (nmol/L)	53.7 ± 0.3	6.0 ± 0.4	23.7 ± 1.0	9.6 ± 0.5	20.7 ± 0.7	41.3 ± 0.6

Note: α-Toc = α-tocopherol, α-Carot = α-carotene, β-Carot = β-carotene, β-cryp = β-cryptoxanthin, Lut/zea = combined lutein and zeaxanthin, Lyco = lycopene. Values of dietary and serum micronutrients are presented as weighted means ± SEs. ^a^ Estimated using Spearman correlation coefficient. ^b^ Participants with metabolic dysfunction-associated steatotic liver disease (MASLD). ^c^ Participants without metabolic dysfunction-associated steatotic liver disease (MASLD).

## Data Availability

The NHANES database is publicly available at https://wwwn.cdc.gov/nchs/nhanes/Default.aspx (assessed on 15 April 2024).
